# Health coaching to promote healthier lifestyle among older people at moderate risk for cardiovascular diseases, diabetes and depression: a study protocol for a randomized controlled trial in Sweden

**DOI:** 10.1186/1471-2458-13-199

**Published:** 2013-03-06

**Authors:** Klas-Göran Sahlen, Helene Johansson, Lennarth Nyström, Lars Lindholm

**Affiliations:** 1 Department of Public Health and Clinical Medicine, Division of Epidemiology and Global Health, Umeå University, SE-901 85 Umeå, Sweden; 2 2Department of Nursing, Umeå University, SE-901 85 Umeå, Sweden

**Keywords:** Life style changes, Prevention, Motivational interviewing, Older people, RCT, Health coaching, Health economics

## Abstract

**Background:**

The challenge of an aging population in the society makes it important to find strategies to promote health for all. The aim of this study is to evaluate if repeated health coaching in terms of motivational interviewing, and an offer of wide range of activities, will contribute to positive lifestyle modifications and health among persons aged 60–75 years, with moderately elevated risk for cardiovascular disease (CVD), diabetes, or mild depression.

**Methods/Design:**

Men and women between 60 and 75 are recruited in four regions in Sweden if they fulfill one or more of the four inclusion criteria.

•Current reading of blood pressure (140-159/90-99) without medication.

•Current reading of blood sugar (Hba1c 42–52 mmol/mol) without medication.

•A current waist-circumference of ≥94 cm for men and ≥80 for women.

•A minor/mild depression (12–20 points) according to Montgomery-Åsberg Depression Rating Scale without medication.

Individuals with a worse result than inclusion criteria are treated according to regular guidelines at the PHCs and therefore not included. Exclusion criteria for the study are dementia, mental illness or other condition deemed unsuitable for participation.

All participants fill out a questionnaire at baseline, and at the 6-, 12- and 18-month follow-ups containing questions on demographic characteristics, social life, HRQoL, lifestyle habits, general health/medication, self-rated mental health, and sense of coherence. At the 12-month follow-up, the health coach will give each participant a second questionnaire to capture attitudes and perceptions related to health coaching and venues/activities offered.

Qualitative data will be collected twice to obtain a deeper understanding of perceptions and attitudes related to health and lifestyle/lifestyle modifications. A health economic assessment will be performed. Individual costs for health care utilisation will be collected and QALY-scores will be estimated.

**Discussion:**

Several drawbacks can be identified when conducting research in real life. However, many of the identified problems can diminish the positive results of the intervention and if the intervention shows positive effects they might be underestimated.

**Trial registration:**

Current Controlled Trials ISRCTN01396033.

## Background

In 2025, one third of the Swedish population will be 60 years or older; in 2050, the average life expectancy is predicted to rise to 86.2 years for women and 83.6 years for men [1]. A higher proportion of the elderly may increase the demand for health care and elderly care [2].

In Sweden, the county councils/regions (n=21) are responsible for health care while the municipalities (n=290) are responsible for elderly care. There is no hierarchical relation between counties/regions and municipalities since they each have their own self-governing local authority with responsibilities for different activities.

Elderly care and health care are fundamental to the Swedish welfare model. They are publically financed, available to all according to needs. Elderly care is provided through a wide range of services, including home services, sheltered houses, housing adaption, transportation services, etc.[3], while health care is provided mainly by hospitals and primary health care. Half of the county councils/regions budget is allocated to hospital care and 16% to primary health care. In 2010, Sweden spent 3.2% of the gross domestic product (GDP) [4] for elderly care and 9.7% for health care [5] of which the majority is utilized by older people, similarly to other contexts [6].

The possible resource savings in health care and elderly care through health promotion and preventive activities are great, and in the short term likely to be even greater for older than for younger persons due to the fact that the older use more resources [2,7]. The demographic transition is a challenge regarding the need of health care and elderly care, and that challenge implies a great potential for improvement.

A key objective of Swedish national public health policy is to give the elderly the chance to live a good life with high quality. Public health policy also emphasizes that effective public health work is based on a shared responsibility between the public and non-profit sectors. Collaboration increases the possibilities to develop more and better ways to understand and influence health [8]. Accordingly, it is crucial for counties, municipalities and the non-profit sector to invest in health promotion activities. Improving the conditions for the development of better health promotion can lead to increased well-being and less disability in older people, and the need for care can be postponed.

The challenge of an aging population in the society led to the government commissioning the Swedish National Institute of Public Health (SNIPH) to develop and manage a partnership model for healthy aging. Collaboration amongst county councils, municipalities and non-profit organizations is the foundation for the partnership model. The goal is to facilitate collaboration on different activities that will help older people with mild illness to change their lifestyle. A specific part of the mission is to launch an intervention directed to the target group aged 60–75 years with moderately elevated risk for cardiovascular disease (CVD), diabetes, or mild depression. Through repeated health coaching sessions and opportunities to visit venues and participate in various physical, social and cultural activities, participants will be supported to better health through lifestyle modifications. An independent research-based process and outcome assessment will be made of this intervention.

This study protocol describes the design of the intervention, with a focus on the outcome assessment.

### Aim

The aim of this study is to evaluate if repeated health coaching in terms of motivational Interviewing (MI) [9] will contribute to positive lifestyle modifications and health among persons aged 60–75 years, with moderately elevated risk for cardiovascular disease (CVD), diabetes, or mild depression. The specific aims are to estimate the impact of the intervention on the health-related quality of life (HRQoL), clinical parameters and measurements (especially blood pressure, HbA1c and waist circumference), self-reported lifestyle habits and whether the intervention is cost-effective.

## Design/Methods

### Institutional responsibilities and intervention

Four county councils/regions with a total of 17 associated municipalities are included in the intervention. The intervention may be described as a partnership for healthy aging. The partnership refers to the local primary health care center (PHC) and the municipality as each of them is responsible for one part of the project: the PHC for the health coaching and the municipality for the organization and responsibility for local venues.

In addition, SNIPH and the non-profit sector each have their roles but share the same goal, to promote health and facilitate lifestyle modification from a holistic perspective.

### The Swedish National Institute of Public Health (SNIPH)

SNIPH is responsible for management, education, and financial issues of the intervention. To increase the willingness of the county councils/regions to participate, the SNIPH allocated 15,000 SEK (1750 Euro) per participant in the intervention group and 3000 SEK (350 Euro) per participant in the control group, to cover all costs including intervention management costs.

**Figure 1:**
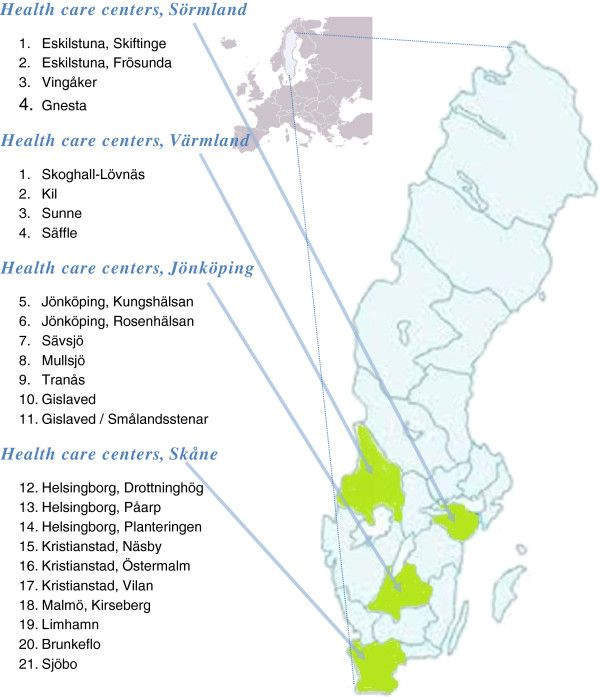
Selected county councils/regions and included health care centers.

Communication with the government and recruiting stakeholders started in 2010. The recruitment of four county councils/regions interested in preventive activities was finalized during spring 2011: they are Skåne, Jönköping, Sörmland and Värmland (Figure 1). These county councils/regions were selected from different parts of Sweden to include different socio-cultural living conditions.

In order to prepare for the intervention, SNIPH carried out educational activities in each county council/region, inviting personnel from both the PHCs and the venues. The goal was to gain a mutual understanding on concepts and methods used in the intervention, and on how active and healthy aging can be promoted. Main topics at the educational activities were inclusion criteria, measurements, the roles of different actors, learning more about MI and stimulating the development of venues and related activities.

### The county councils/regions

In collaboration with the municipalities, the county councils/regions selected a total of 21 PHCs (Figure 1). In each county council/region, one project manager is responsible for the activities operated by the PHCs, and for carrying the collaborative work forward together with the municipalities. The selection of PHCs was made to represent both urban and rural areas as well as different socio-economic structures.

The PHC is responsible for recruiting health coaches with suitable educational qualifications and experiences, e.g. physiotherapists or community health nurses. Furthermore, the PHC is in charge of recruiting participants to the intervention. Methods for recruiting are advertising, information given at the PHCs, active outreach in public places, such as information to senior organizations, and letters of invitation to participate generated from patient records. The PHC is also responsible for initial screening, randomization to the control group or the intervention group, follow-up measurements and recording and storing data.

All participants have access to regular primary care. Participants in the intervention group are offered repeated health coaching sessions based on MI, including recommendation and motivation to participate in different physical, social and cultural activities. Persons developing high blood pressure or diabetes during the intervention period will be treated following available practices at each PHC. Test results are communicated to the participants in the intervention group during the MI session and to participants in the control group by the ordinary staff at the PHC.

The role of the health coaches is by MI, to motivate and support participants to modify their lifestyle. The completed questionnaires and the results from the measurements should be used as points of departure for the MI sessions.

The health coach and the participant meet at baseline and after 3, 6 and 12 months. In between these planned meetings, opportunities will be given for additional supporting conversations though extra meetings or telephone calls to increase the likelihood of lifestyle modifications.

In addition to MI, the participants will be offered and, depending on the individual motivation, encouraged to participate in activities available at venues operated by municipalities and non-profit organizations. Health coaches have enough knowledge about venues in the surrounding area to help participants find a suitable type of activity.

MI is an evidence-based approach built on cooperation between the interviewer and the individual. Individual motivation or resistance to events such as lifestyle changes plays a significant role in health and can be influenced by the interviewer's conversation strategies. The use of MI is growing rapidly, and there is strong evidence that MI is an effective treatment, especially with alcohol, tobacco and drug abuse but also in other behavioural changes. MI is more effective than traditional counselling, and MI effects are more prolonged and stable than other interventions ([10]). Furthermore, MI is regarded as cost-effective ([11]).

To ensure the quality of the interview technique, all health coaches were offered five MI conversations spread over 18 months and coded according to Motivational Interviewing Treatment Integrity Code 3.1. (MITI) [12]. The coaches sent in a tape of 20 minutes of MI conversation to Miclab [13], which conducted the coding. The MITI is a behavioural coding system which includes coding of twelve variables and estimates how well the MI is applied by the health coach. This method is used both as a treatment integrity measure of MI and to provide structured, formal feedback to the practitioner.

### The municipalities

The municipalities are responsible for organizing venues. The venues should offer health promoting activities that meet the participant’s interests and need. A venue can either be operated by the municipalities or by non-profit organizations. Four factors are important to promote healthy aging (known as the ‘four cornerstones’); physical activity, healthy eating habits, social interaction/support and participation/meaningfulness/feeling. These four factors can affect health, especially for those who are still independent [14]. Hence, the requirements for venues are opportunities for physical activities, activities related to food and cooking, as well as cultural and social activities. The social activities should promote the creation of new relationships and fellowship among the participants and can vary depending on local conditions. One municipality has identified 43 venues, among which are found the city library, the art museum, yoga sessions, gyms, hydro-gymnastics, health lecturers and cooking courses. Another municipality, though, offers only three specific activities for participants.

Each municipality has a local coordinator who has the overall knowledge about the venues/activities offered by the municipalities as well as by non-profit organizations. The local coordinators are responsible for the development of venues and interaction with the stakeholders.

### The non-profit sector

Non-profit organizations have no particular area of responsibility, but since they already run a range of physical and social activities, their involvement is considered necessary to meet the needs of the target population. Through collaboration with the municipalities, the non-profit sector´s expertise may be used to add resources in health promotion among the elderly.

### Study population

The financial framework provided by the government allowed recruitment of 2000 participants, of which 1500 were assigned to the intervention group and 500 to the control group.

The target group comprises persons aged 60–75 years with early signs of hypertension, diabetes, obesity or mild depression. The following four inclusion criteria were applied:

1. Current reading of blood pressure (140-159/90-99) without medication.

2. Current reading of blood sugar (Hba1c 42–52 mmol/mol) without medication.

3. A current waist-circumference of ≥94 cm for men and ≥80 for women.

4. A minor/mild depression (12–20 points) according to Montgomery-Åsberg Depression Rating Scale (MADRS) [15,16] without medication.

Individuals with a worse result than inclusion criteria are treated according to regular guidelines at the PHCs and therefore not included. Exclusion criteria for the study are dementia, mental illness or other condition deemed unsuitable for participation.

### Randomization

The randomization procedure was done in two steps. First, each PHC centre received a list with code (ID) numbers where each number was assigned to either intervention or control group by using Excel software. By random assignment each included participant was given an ID number and allocated to the corresponding group. The total number of codes for each PHC differed, depending on the number of participants planned to recruit, but the distribution between intervention and control group were the same.

### Data collection

#### Measurements

**Figure 2:**
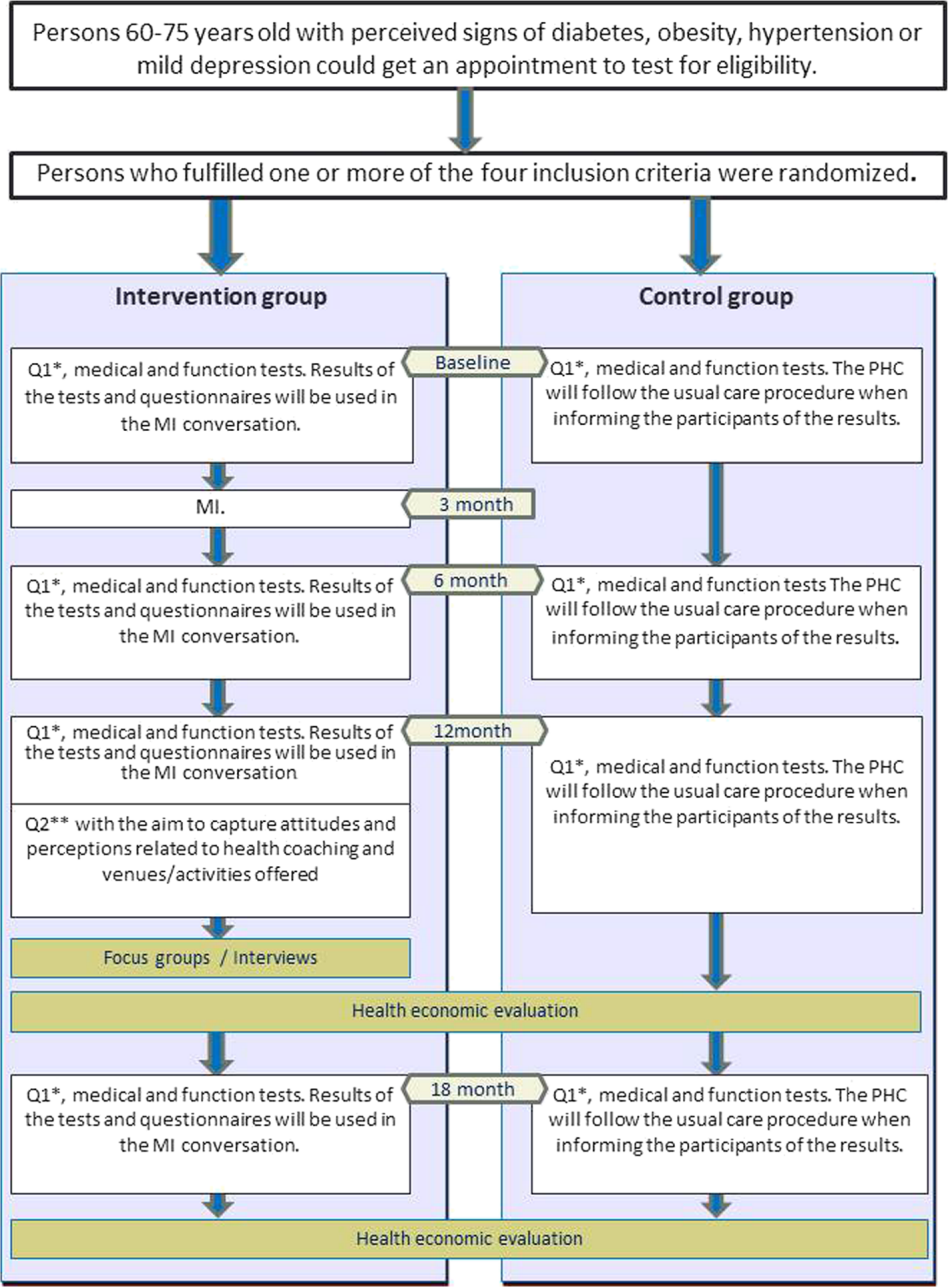
Timing of data collection and measurement instruments used.

At baseline and at 6, 12 and 18 months (Figure 2), the following measurements will be made of all participants according to the standardized procedures shown in Table 1: blood pressure, waistline, weight, blood sugar level, cholesterol levels, resting heart rate and 6-Minute Walk Test [17]. Height will only be measured at baseline.

**Table 1:** Standardized procedures of measurements

**Blood pressure(BP)**	• Measured manually or digitally
	• Measure BP after 5–10 minutes of rest. The individual should avoid coffee, tobacco or heavy physical activity 30 minutes prior to the measurement.
	• The participant should sit comfortably with the back supported and the feet on the floor (no crossed legs).
	• Use a cuff with adequate width.
	• For manual measurement, pump up and palpate first the systolic BP to avoid an error record
	• (auscultatory gap). Release the air from the bladder with a speed of 2–3 mm Hg/pulse beat. Empty the bladder completely between registrations.
	• Use the average of at least two registrations with one minute intervals and read at the nearest even number (to avoid digit preference). For large variations (>5 mm Hg); use the average of more registrations.
	• Measure in both arms the first time and choose the arm with highest pressure (difference>10 mm Hg) for further measurements. If the pressure does not vary, use the right arm.
**Waist measurement**	• Select a point about 2 cm above the belly button (just below the lower rib cage).
	Measure with tape after a relaxed exhalation. Make sure the tape has not "hooked" up in the lower back.
	• Read throughout cm.
**Weight**	• Use a calibrated digital scale for medical use.
	• The weighing should be done at the same time, preferably in the morning before breakfast.
	• Participants must remove shoes.
	• Round off to the nearest 0.1 kg.
**Height**	• Measured in cm, standing fully upright, without shoes or headgear. The back should rest against a wall and the head should be forward.
**Blood samples**	• The following samples should be taken: LDL cholesterol, HDL cholesterol, triglycerides, HbA1c.
	• Must be preceded by a 10-hour fast (water allowed).
	• Before sampling, the skin must be cleaned with disinfectant.
	• Blood samples are taken with a needle from the vein in the antecubital area.
**6**-**Minute Walk Test**	• The test is performed in a corridor on a 30 m distance with as little distraction as possible.
	• Mark the starting line on the floor with tape. The distance is then marked every 3 meters, preferably on the wall.
	• Repeated tests should be done about the same time of the day to minimize diurnal variation.
	• Participants should wear comfortable clothing and appropriate footwear. Walking aids, which are normally used, are permitted.
	• The participant should not have done strenuous work two hours before the test.
	• Before the test starts, resting heart rate and blood pressure should be measured). Resting heart rate is noted.
	• The test includes the estimation of the degree of exertion using the Borg RPE scale [18] and instructions for this are given before the test.
	• The instruction to the participant are; go as far as possible during 6 minutes (do not jog or run). Walking speed is up to the participant but the goal is to become exerted by going back and forth. It is allowed to reduce speed or stop and rest (leaning against the wall is allowed), but the clock is running. If the participant for any reason must stop the test, the total walking distance is measured.
	• Avoid talking during the test. Do not walk together with the participant.
	• Measure the length of the walk by counting each time the participant returns to the starting line.
	• Notify the participant when 3 minutes, 1 minute and 15 seconds remain.
	• After the test, record heart rate, estimate the degree of exertion and calculate the total walking distance rounded to whole meters.

### Questionnaires

All participants fill out a questionnaire (Q1) at baseline, and at the 6-, 12- and 18-month follow-ups containing questions on demographic characteristics (only assessed at baseline), social life, HRQoL (EQ5D) [19,20], lifestyle habits, general health/medication, self-rated mental health (MADRS) [16,21], and sense of coherence (SOC-13) [22-24] (Table 2).

**Table 2:** Questionnaire areas with associated variables and references

**Area**	**Variable (reference)**
Demographic characteristics	Gender, age, country of birth, educational level, employment, sick leave/pension
Social life	Participation in different organizational and societal activities, possibilities to get emotional and/or financial support [25]
HRQoL	EQ 5D-questionnaire: a five-item questionnaire covering five dimensions of health and a visual analogue scale (VAS)
Lifestyle habits	10 questions assessing tobacco use, alcohol consumption, eating habits, physical activity [26]
General health and medication	Questions on general health and current use of drugs, modified version [25]
Self-rated mental health (depression)	Montgomery-Åsberg Depression Rating Scale (MADRS): 9-item self-assessment version [15,16]
Sense of coherence	Antonovsky’s Sense of Coherence Scale: 13-item short version (SOC-13) [22,24]

At the 12-month follow-up, the health coach will give each participant a questionnaire (Q2) and a pre-paid return envelope addressed to the research group. The aim of Q2 is to capture attitudes and perceptions related to health coaching and venues/activities offered. The participants are instructed to fill out the questionnaire at home and send it by surface mail.

### Focus group discussions (FGDs)/in-depth interviews

Qualitative data will be collected twice: at the beginning of the intervention and after the last MI at the 18-month follow-up. This is mainly to obtain a deeper understanding of perceptions and attitudes related to health and lifestyle/lifestyle modifications, as well as experiences of health coaching and venues. Focus group discussions will be held only in the intervention group.

One PHC in each county will be selected, guided by the pursuit of variation (urban/rural, high/low percentage of immigrants, women/men, etc.). Focus group discussions will be supplemented by a number of in-depth interviews. The study adheres to the RATS guidelines.

### Health economy

A health economic assessment will be performed. Costs for the intervention will be gathered from counties/regions and municipalities. Individual costs for health care utilisation will be collected from medical records and registers. QALY-scores are estimated for each individual based on their answers in the questionnaire and the cost per QALY gained will be estimated.

### Data management

A prepared Excel document is delivered to every PHC. For each individual, a personal ID is created from the county ID, the PHC ID and a random individual ID. All ID numbers belong to either the control group or the intervention group. All data transferred from the PHC to the research group are anonymous.

The Excel document is organized to store one sheet for each period of data collection. Every cell in the document is prepared to minimize errors. Data collected at baseline are more comprehensive than the following as they contain background characteristics of the participants.

### Outcome measures

The possible effect of lifestyle changes will be estimated for participants fulfilling each of the four inclusion criteria. Thus, primary outcome variables are blood pressure, HbA1c, waist circumference and the MADRS scale [16,21]. Effects on participants fulfillingq more than one criterion will also be analysed. Independently of inclusion criteria, the health-related quality of life will be followed over time using the instrument EQ-5D [19] as well as the clinical parameters triglycerides, HDL and LDL.

In addition, we will analyse some participant performance indicators related to the four cornerstones for healthy life [14]. These indicators are the validated SOC questionnaire [24], self-assessed frequency of physical activity, 6-minute walk test, and self-assessed frequency of eating habits.

### Data analysis

The difference between measurements at baseline and at the 6-, 12- and 18-month follow-ups will be calculated, and the estimates in the intervention groups will be compared with the controls. Time trend analysis will be applied to test for a possible trend pattern.

EQ5D score is assumed to be 0,8 in the intervention group and 0,75 in the control group, and the standard deviation is assumed to be 0,3 in both groups. The intervention groups should be three times larger than the control group, power equal to 0,8 and alpha equal to 0,05. A two-side test requires thus 1132+377 = 1509.

Interviews and FGDs will be analysed using qualitative content analysis [27].

### Ethics and trial registration

All participants are informed about the purpose of the study. Written informed consent is obtained and participants are ensured confidentiality. They are all informed about the possibility to end their participation whenever they want. All data are collected at each PHC and handled with secrecy. All participants are given a unique ID number which is sent together with data to the research group. The Regional Ethical Review Board in Umeå, Sweden, approved the study in August 2011, ref: Dnr 2011-230-31. The intervention is registered at the Current Controlled Trials web page (ISRCTN01396033).

## Discussion

To do research in real life settings is challenging. There are a number of problems and drawbacks that are important to be aware of that might introduce problems in drawing definite conclusions.

It is time-consuming and difficult to educate and instruct health coaches in more than 20 PCC units. In total, at least 100 staff persons are involved in the intervention. We have tried to write a concrete and comprehensive manual to cover all questions regarding how to recruit participants, how to get informed consent, how to randomize participants, how to register the questionnaires, how to make the medical and functional tests, how to register the test results and how to send the data to the research group. However, during the process, new questions have arisen, and even topics that are covered in the manual have not always been understood properly. These factors may introduce error in the results.

MI is a relatively well-known and widespread method that is used as a tool in intervention as well as part of”usual primary care”. This may reduce differences between the control group and the intervention group. However, in the present study, the health coaches dispose radically more resources in the intervention group than a usual PHC does for participants in the control group.

County councils/regions that initially were invited to participate in this intervention were characterized as being in the forefront among Swedish counties with regard to preventive actions. This selection may diminish the potential of the method since other county councils/regions might have more to gain by implementing a successful strategy.

The staffs at the PHC are continuously informed on the importance of lifestyle changes that might affect ordinary work and thus also affect the control group. This may reduce the differences between the intervention group and the control group and thus diminish a possible positive result.

Controls were selected at the same PHC unit, implying that they are living in the same context as those who get the MI intervention, but this can also introduce a couple of problems. Firstly, some of the controls might be affected by neighbours and relatives who get the intervention. Secondly, when a person in the control group makes progress (as a result of the intervention tests) and receives care according to current procedures and care programs at the PCC unit, this may be regarded as an intervention in itself. The comparison between groups is supposed to be between regular care and intervention, but it could be a comparison between two interventions as even the control group receives intervention in the form of testing and questionnaires. Both problems will reduce the differences between the two groups and hence positive effects may be underestimated.

The intervention comprises MI and the described tests and questionnaires. In addition, municipalities offer social meeting points. They can be organized and carried through differently in different settings. If one health coach can refer to a successful meeting point, it can be interpreted as a positive way of working with the MI. However, this will not affect the differences between the two groups, since both groups will gain or suffer from the same local differences.

## Abbreviations

BP: Blood pressure; CVD: Cardiovascular disease; EQ5D: EuroQol 5 dimensions; GDP: Gross domestic product; HRQoL: Health-related quality of life; MI: Motivational Interviewing; MADRS: Montgomery-Åsberg Depression Rating Scale; SNIPH: Swedish National Institute of Public Health; SOC: Sense of coherence; VAS: Visual analogue scale.

## Competing interests

All authors declare that they have no competing interests.

## Authors’ contributions

All authors have made substantial contributions to this manuscript and the final version is a result of several discussions and team work among the authors. KGS, LL, LN, and HJ worked together with the study design. KGS made the first draft of the paper and HJ made substantial contribution to the draft. LN and LL contributed to the final version by writing and structuring the article. LL developed the health economic part. LL, LN, HJ, and KGS all approved the final version.

## Pre-publication history

The pre-publication history for this paper can be accessed here:

http://www.biomedcentral.com/1471-2458/13/199/prepub
